# The Role of Thyroid Hormones as Inductors of Oxidative Stress and Neurodegeneration

**DOI:** 10.1155/2013/218145

**Published:** 2013-12-09

**Authors:** I. Villanueva, C. Alva-Sánchez, J. Pacheco-Rosado

**Affiliations:** Departamento de Fisiología, Escuela Nacional de Ciencias Biológicas, IPN. Prol. Carpio y Plan de Ayala, s/n, 11340 México City, DF, Mexico

## Abstract

Reactive oxygen species (ROS) are oxidizing agents amply implicated in tissue damage. ROS production is inevitably linked to ATP synthesis in most cells, and the rate of production is related to the rate of cell respiration. Multiple antioxidant mechanisms limit ROS dispersion and interaction with cell components, but, when the balance between ROS production and scavenging is lost, oxidative damage develops. Many traits of aging are related to oxidative damage by ROS, including neurodegenerative diseases. Thyroid hormones (THs) are a major factor controlling metabolic and respiratory rates in virtually all cell types in mammals. The general metabolic effect of THs is a relative acceleration of the basal metabolism that includes an increase of the rate of both catabolic and anabolic reactions. THs are related to oxidative stress not only by their stimulation of metabolism but also by their effects on antioxidant mechanisms. Thyroid dysfunction increases with age, so changes in THs levels in the elderly could be a factor affecting the development of neurodegenerative diseases. However, the relationship is not always clear. In this review, we analyze the participation of thyroid hormones on ROS production and oxidative stress, and the way the changes in thyroid status in aging are involved in neurodegenerative diseases.

## 1. Introduction

Reactive oxygen species (ROS) are oxidizing agents amply implicated in tissue damage. ROS are originated from both exogenous and endogenous sources, and the respiratory redox chain in the mitochondria is considered the major source of ROS and free radicals in the cell. This implies that ROS production is inevitably linked to ATP synthesis in most cells. ROS and other related radicals serve diverse physiological functions in the cell [1, 2] but can also react unspecifically with cell components, thus reducing their functionality and causing oxidative damage. The excess ROS and free radicals are normally eliminated by antioxidant mechanisms comprising enzymatic and nonenzymatic radical scavenging and neutralizing systems. It has been hypothesized that the organic alterations associated to aging and to some chronic diseases would originate from the accumulation of punctual modifications in the mitochondrial DNA caused by mild oxidative damage over the lifetime of the organism. In turn, this would lead to a progressive reduction of the electron transfer efficiency in the respiratory machinery and thus to gradual increase in the rate of ROS production and oxidative damage [3–5]. Both the rate of ROS production and the activity of the radical-eliminating systems vary according to diverse factors ranging from energetic demand of the cell to the expression rate of specific genes.

## 2. Metabolic Rate and ROS Production

Numerous cellular enzymatic processes in the cytosol, the endoplasmic reticulum, peroxisomes, and the inner and the outer mitochondrial membranes generate ROS [6]. From these subcellular locations, the inner milieu of the mitochondria is considered the major ROS-producing compartment in metabolically active tissues [7]. Normally, these ROS do not permeate to the cytosol, being disposed of locally [8]. From the ROS originated in various mitochondrial reactions, the main proportion arises from the respiratory chain: a sequence of redox reactions that channel electrons from the reducing NADH or succinate to the final acceptor oxygen. The electron transfer is coupled to proton pumping from the mitochondrial matrix to the intermembrane space, thus originating an electrochemical gradient in which the intermembrane space becomes positively charged in relation to the matrix. The gradient provides the energy to bind phosphate to ADP and yield ATP. Most cellular ROS production is then linked to the aerobic ATP synthesis. The relation between respiratory rate (measured as the volume of O_2_ consumed per min) and ROS production is not a direct one, for low respiratory activity is often associated with high ROS generation, whereas the raise in the activity of the respiratory chain can result in a decrease in the rate of ROS production. This paradoxical relation derives from the fact that the electron transfer in mitochondria can proceed at different paces according to the availability not only of oxygen but also of energetic substrates and ADP [9], which in turn depend on the general energetic state of the cell. ROS formation is initiated by the diversion of an unpaired electron to O_2_ or other acceptor species in some intermediate steps of the respiratory chain, particularly the complexes I and III (the “normal” reduction of O_2_ to form H_2_O occurs in the complex IV). The electron diversion to ROS precursors is favored by conditions that retard or reverse the downhill electron flux through these complexes, for instance, a highly reduced state of the respiratory carriers, a reduced ADP availability (high ATP/ADP ratio) [10], a high electrical potential of the matrix [11], and a relative disorganization of the respiratory-complex clusters in the mitochondrial membrane [12]. When the cell activity raises, the increase in energy expenditure implies an increase in the rate of ATP breakdown that leads to augmented ADP availability and an oxidative state represented by a low NADH/NAD^+^ ratio (state 3). Both ADP and oxidative state stimulate the electron flux through the respiratory chain until the final O_2_ reduction in complex IV is achieved diminishing the electron transfer in intermediate steps to form ROS. As a result, the production of ROS is more than five times greater when the mitochondria are in the low-rate respiration, ADP depleted state (state 4) than when in the active respiration, maximal oxygen consumption state (state 3) [13, 14] induced by increased energy expenditure.

The ROS formed by diverse processes in the cell are continuously eliminated by means of both enzymatic and nonenzymatic mechanisms. The nonenzymatic elimination of ROS is based on sequestration and neutralization of radicals already formed and involves small water- or lipid-soluble molecules such as vitamin E derivatives, coenzyme Q, lipoic acid, and the tripeptide glutathione. The enzyme-mediated elimination of ROS involves the isoforms of superoxide dismutase, which channel the radicals to the formation of H_2_O_2_ and must then work in concomitance with the H_2_O_2_ removing enzymes peroxidase and catalase. Other two enzymes, glutathione peroxidase and glutathione reductase, catalyze the transfer of radicals mediated by glutathione. A third antioxidant mechanism consists in enzyme-mediated repair or destruction of the molecules and structures damaged by the free radicals [15]. A main feature of the enzymatic mechanisms is that the relative activity of the antioxidant pathway can be regulated by signals related to the oxidative state of the cell, thus allowing compensatory adjustments to the increases in ROS production [16, 17]. Conversely, nonenzymatic scavengers tend to be depleted as ROS production increases, unless compensatory adjustments also accelerate their recycling. As a consequence, the different forms of antioxidant activity can change according to the degree of oxidative stress, but the pattern of change varies. For instance, a rise in the rate of ROS production causes a drop in the availability of antioxidant substrates such as glutathione and vitamin E, while promoting the expression of some antioxidant enzymes [18, 19]. The manifestation of oxidative damage (i.e., accumulation of lipid peroxidation and protein carbonyl products) would depend on the overall efficiency of the antioxidant mechanisms and their ability to cope with higher demands. Finally, different tissues seem to respond differently to the induction of oxidative stress, in both the ROS production and the antioxidant capability [12].

## 3. Thyroid Hormones, Metabolism, and ROS Production

Iodine compounds (IC) are a group of signaling molecules based on the incorporation of a variable number of iodine atoms, typically 1–4, in an organic molecule derived from the aminoacid tyrosine. The main source of iodine compounds in the vertebrate systems is the thyroid gland, which releases to the blood stream significant quantities of tetraiodothyronin (thyroxin, T4) and a much smaller proportion of triiodothyronin (T3). These two compounds exert actions at the cell level by binding a set of specialized receptors that couple to both genomic and nongenomic signaling pathways. Besides these actions, the thyroid hormones (THs) are subjected to a series of transformations in the peripheral tissues, mainly in the form of deiodination but also decarboxylation, that originate diverse derivatives with signaling capacity [20, 21]. These peripheral transformations could even generate local levels of some derivatives that exceed the circulating levels of THs [22].

THs exert a wide series of effects acting upon virtually all tissues in the organism. The actions of the various ICs derived from the THs are not well known and seem to differ significantly. For instance, diiodothyronin (T2) produces metabolic effects similar to those of T3 [23] whereas thyronamines oppose its actions [24], at least at the mitochondrial level. This aspect of the thyroid physiology deserves further investigation. The known actions of the THs can be grossly classified in two general processes: regulation of growth and development and regulation of metabolism. The metabolic effects of THs are directly linked to ROS production and oxidative stress in various ways. First, the general metabolic effect of THs (and IC in general) is a relative acceleration of the basal metabolism that includes an increase of the rate of both catabolic and anabolic reactions [25]. This results in increased energy expenditure, fuel mobilization, fuel oxidation for energy extraction, oxygen consumption, respiratory rate, and heat production and release [26]. The stimulation of the respiratory rate would intuitively lead to greater ROS production but, as noted above, the relation between these two variables is not linear. Instead, ROS production depends largely on the mitochondria being switched between states 3 and 4. Although THs do not directly determine the respiratory state of the mitochondria [27, 28], stimulation by THs promote state 3 by augmenting ATP breakdown by different energy-consuming mechanisms in the cell [26] and thus increasing ADP availability. This would be expected to decrease ROS production. However, THs also promote a reduction state in the cell by increasing fuel availability and extramitochondrial production of ATP and NADH, which in turn promote reduction of the components of the mitochondrial respiratory chain and transition to state 4 [29]. THs have also been shown to stimulate the synthesis of elements of the respiratory chain, which further enhances the reductive state [29–31]. In such a situation ROS production is expected to increase. THs also promote extramitochondrial ROS production by modifying the expression of genes coding for enzymes involved in ROS production and elimination [32, 33]. Finally, the increase in TH levels has been shown to modify the composition of membrane phospholipids [34] increasing the degree of unsaturation particularly in the mitochondrial membranes [35]. Since unsaturation of fatty acids makes them more susceptible to free radical attack [36], this effect results in augmented lipid peroxidation in mitochondria [35]. On the whole, the effect of THs on ROS production varies between tissues according to their specific susceptibility [18].

**Figure 1:**
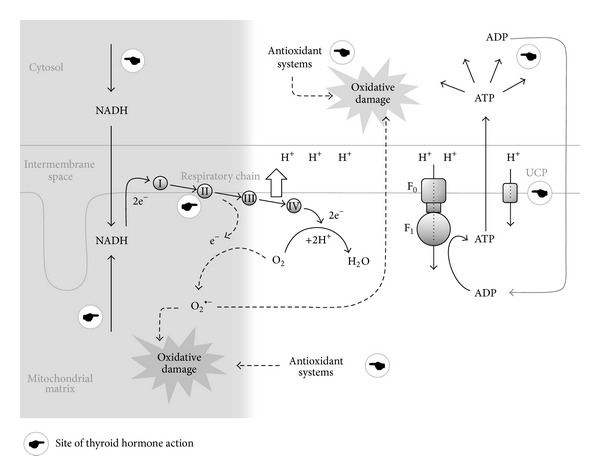
Main pathway of ROS generation in the cell and sites where it is modified by the thyroid hormones. Continuous lines represent the “normal” energy yielding pathway; dotted lines represent the pathways leading to ROS production. The respiratory chain in the internal mitochondrial membrane receives a pair of electrons coming from the oxidation of metabolic fuels and brought to the site by intermediaries (mainly reduced NAD, NADH). The electrons are transferred through an energetic downhill flux to the final acceptor O_2_ to yield H_2_O. The energy extracted from electrons is used to pump protons (H^+^) to the intermembrane space. The proton gradient that builds up powers the proton flux through the ATP-synthase complex (F_1_F_0_) which drives ADP phosphorylation to produce ATP. ATP provides energy for cell reactions where it is broken down to ADP plus phosphate. Unpaired electrons can divert from this pathway in an intermediate step of the respiratory chain and combine with other species, mainly O_2_, to form the superoxide anion (O_2_^∙−^). Further reactions produce highly reactive radicals that combine with and alter structural and functional elements of the mitochondria, thus producing local oxidative damage. The radicals can permeate outside the mitochondrion and cause cell oxidative damage. Both mitochondrial and cytosolic antioxidant systems scavenge and neutralize radicals and destroy or repair damaged elements. The shaded area in the left includes the processes promoting ROS formation. These favor electron diversion by “pushing” electrons through the respiratory chain (i.e., state 4). The processes in the right reduce the diversion of electrons by “pulling” them from the end side of the pathway (i.e., state 3), thus reducing ROS formation. Thyroid hormones (THs) stimulate both ROS-producing and ROS-reducing processes (from left to right): they favor a reductive state by promoting the oxidation of fuels to produce NADH and extramitochondrial ATP (with depletion of ADP). They also stimulate the synthesis of elements of the respiratory chain, which enhances the reductive state. On the other hand, THs act as radical scavengers and promote the expression of antioxidant enzymes, thus decreasing the oxidative damage. The general metabolic activation caused by THs increases the ATP breakdown and raises ADP availability. Finally, the dissipation of the proton-motive force by means of the uncoupling proteins (UCP) decreases the electron diversion and the formation of ROS. UCP genes are targets of the THs.

The THs also affect the cell antioxidant status. In the first instance, due to the chemical properties derived from their molecular structure, diverse IC can act as free radical scavengers and reduce oxidative damage in biological preparations [37, 38]. These antioxidant actions are independent of the receptor-mediated effects of the hormones, and their relative contribution to the general antioxidant status is not clear. The receptor-mediated actions of the THs involve a general effect of raising the levels of nonenzymatic free radical scavengers [39], which simultaneously tend to be depleted by the increased radical concentration. As for the activity of antioxidant enzymes, the effect of TH stimulation varies amply depending on the specific enzyme, the tissue assayed, and the degree of stimulation. In general terms, the activity of some enzymes, such as superoxide dismutase, increases under TH stimulation along with the rate of ROS production. Other enzymes such as catalase and glutathione peroxidase are controlled differently and can be reduced [18, 40] or augmented by TH stimulation. On the other hand, a reduction of the TH activity involving decreased ROS production (as in hypothyroidism) depresses the antioxidant activity, both enzymatic and nonenzymatic [41–43]. Since this condition also involves reduced ROS production, the decline in the antioxidant capacity does not necessarily result in oxidative stress. Finally, besides the classical antioxidant enzymes, other proteins could also participate in the modulation of oxidative stress by THs. This is the case of the uncoupling proteins (UCPs), a family of pore forming channels that favor the proton leakage from the intermembrane space to the matrix of the mitochondria, thus reducing the electrochemical gradient that powers the ATP synthesis. By reducing the negative potential of the matrix, UCPs reduce the possibility of electrons being diverted from the respiratory path and transferred to ROS precursors. Although the main function traditionally attributed to the UCPs is the energy dissipation in the form of heat for temperature and body weight regulation [44], their presence in tissues not involved in these functions [45], in animals not regulating their body temperature, and even in unicellular organisms suggests a critical involvement (also) in the regulation of ROS production [46]. Based on the fact that the UCP genes are targets of the genomic effects of the THs [47], UCPs could be considered among the antioxidant nonenzymatic mechanisms promoted by THs. A general scheme of the TH actions on the pathways of production and elimination of ROS is presented in Figure 1.

The fact that the THs affect simultaneously various aspects of the oxidative stress, inducing different and even opposite effects, could explain the inconsistencies in the reports on the effects of hypo- and hyperthyroidism on oxidative stress found in the literature. For instance, hypothyroidism has been reported to do not modify [39, 42, 52], to reduce [41, 79], or to increase [43] oxidative damage in metabolically active organs. On the other hand, hyperthyroidism has also been found to increase the levels of lipid peroxidation products in metabolically active tissues [39, 52] and erythrocytes [79] but also to reduce the levels of protein adducts [53] or to produce no significant effects on the indexes of oxidative stress [18] in liver. A situation in which hyperthyroidism reduces oxidative stress while hypothyroidism increases it (i.e., the opposite to the general tendency) has even been found in mouse liver [51]. A summary of representative studies relating thyroid status and parameters of oxidative stress in different species is presented in Table 1. On the whole, the inconsistencies among different studies can be attributed to the hormonal treatment employed (dosage, route of administration, duration, and strategy for inhibition of the thyroid gland) to the species studied and to the tissue assayed [51]. In spite of the discrepancies, some general principles on the effects of THs on oxidative stress can be withdrawn: (1) the metabolic stimulation caused by THs implies an increase in ROS production, related to but not directly derived from the increase of the respiratory rate (i.e., O_2_ consumption); instead it seems to depend on the metabolic state of the mitochondria (state 3 versus state 4). (2) The general balance that results from the stimulation of both production and elimination of ROS by THs implies a net increase in oxidative stress, as measured by cellular damage products such as lipid peroxidation. (3) The degree of oxidative stress promoted by THs varies amply among tissues, with the general principle that the cell types more metabolically responsive to THs (such as liver, heart, red oxidative muscle fibers, and lymphoid tissue) are more affected than the less responsive or anaerobical (i.e., white glycolytic muscle and spleen). (4) Hyperthyroidism implies an increase in oxidative stress that grossly relates to the degree of thyroid overactivity; hypothyroidism implies a reduction of ROS production but also of the antioxidant activity, resulting in nonmodified to reduced (lower than control) oxidative stress. (5) The clinical observations in humans match the mechanisms described by experimental manipulation in rodents.

## 4. Thyroid Hormones and ROS in the Brain

The neural tissue shows a very high respiratory activity that may exceed several times that of other metabolically active peripheral tissues such as liver [12]. Based on classical studies, it is commonly accepted that the respiratory rate of the brain tissue is not affected by THs [25] in spite of having numbers of TH receptors similar to other TH responsive tissues such as liver [91]. More recent studies have found that the induction of hypothyroidism has a depressive effect on different aspects of brain metabolism. For instance, rats with reduced thyroid activity show decreased glucose utilization [92] and reduced activity of the highly energy-consuming Na^+^/K^+^-ATPase in various brain regions [93]. As for mitochondrial respiration, hypothyroidism has been demonstrated to reduce the respiratory rate of mitochondria isolated from neonatal [94] and adult [28] rat brain. The intensity of this reduction varies from mild, nondetectable to more than 30% of the control, depending on the substrate employed and the state induced to mitochondria during the tests. The administration of THs to hypothyroid or euthyroid animals has the general effect of increasing the respiratory rate of the brain mitochondria, raising it significantly above the control if hyperthyroidism is induced [19, 28, 94]. The effect of THs on brain metabolism is attributed to the long-term genomic actions of the THs on the transporter proteins of the mitochondrial respiratory chain [28]. Since THs are known to elicit also short-term nongenomic effects [95], the possibility remains that the metabolic activity of the brain tissue was modified still further *in vivo* by the nongenomic actions of the THs. As far as we know, no data are available in the literature assessing this last issue. Finally, also the activity of the antioxidant mechanisms is affected similarly in brain and peripheral tissues [19, 27]. In sum, the experimental evidence favors the concept that the metabolic activity of the brain is sensitive to TH stimulation similar to other metabolically active tissues.

According to the general principles described above, the elevation of TH activity in the brain is expected to be associated with increased oxidative stress, and hypothyroidism is expected to confer a certain degree of neuroprotection against it. In fact, it has been shown that hypothyroidism implies a reduction in overall oxidative stress as measured by the production of markers of cell damage (i.e., lipid peroxidation and protein carbonylation), whereas hyperthyroidism increases oxidative stress in similar terms [19, 27]. In accordance with the prolonged lifespan of neurons, brain mitochondria are relatively resistant to the induction of ROS production and to the oxidative stress [12]. However, once generated, the oxidative stress provokes considerably more cell damage in brain than in other tissues [96], probably due to particularities in lipid composition or antioxidant mechanisms. This underscores the participation of THs and probably other ICs in the generation of oxidative stress in brain and implicates the thyroid status of the individual as a possible contributing factor in the development of neurodegenerative diseases and cognitive alterations associated with aging.

## 5. Thyroid Hormones and Aging: Clinical Correlation

In both human and animal models aging is associated with a higher prevalence of thyroid disorders. In the elderly, the prevalence of subclinical hypothyroidism reaches 20%, while that of subclinical hyperthyroidism ranges between 2 and 8% [97, 98]. Furthermore, several studies in healthy older individuals have revealed an age-dependent decline in serum TSH and free T3 (FT3) along with an increase in reverse T3 (rT3) and a maintenance of stable serum free T4 (FT4) levels [99–101]. According to these data, aging is expected to be associated with a high incidence of cognitive and mood disorders derived from thyroid dysfunctions. It has been suggested that old patients (>60 years) being diagnosed with subclinical hypothyroidism have an increased risk for depression and that this risk is even greater in cases of overt hypothyroidism [102]. However, the clinical evidence does not clearly show an association between hypothyroidism and mood impairment in the elderly (for review see [103]).

Despite a relatively low prevalence of overt (1-2%) or subclinical hypothyroidism (3–16%) in elderly patients [104], there is the idea that some detrimental traits of aging, such as increased cardiovascular risk, reduced bone density, and cognitive decline, could be related to thyroid impairment, characterized by an elevation in TSH. Both overt and subclinical hypothyroidism have been linked to increased oxidative stress and protein oxidation in adult patients [68, 76]. A strong relation between oxidative stress biomarkers and increased TSH can be established only for cardiovascular risk, associating secondary hypercholesterolemia to hypothyroidism. However, no significant correlation was observed after controlling the total cholesterol levels, which indicates that hypothyroidism *per se* is not causative of oxidative stress in subclinical hypothyroid patients [64]. Studies on the relationship between TSH levels and cognitive function in elderly patients also show contradictory results. Whereas some authors found an inverse relationship between higher TSH levels and poorer cognitive function [105], others found a direct correlation between TSH levels and cognitive performance [106].

THs are known to be necessary for the maintenance of optimal cognitive ability in adults. The relationship between cognitive performance and thyroid status has been established by experimental, clinical, and epidemiological studies. For instance, it has been repeatedly reported that hypothyroidism induced in adulthood provokes cognitive impairment evidenced as a poor working memory and the inability to concentrate on complex mental work [107–109]. Alterations that imply inadequate transport of T3 and T4 into the brain cause impairment of some neural functions, affecting cognition and emotion. In patients with primary thyroid disorders, both hyper- and hypothyroidism can induce behavioral abnormalities that mimic depression, mania, and dementia, and these neuropsychiatric impairments are generally reverted following return to euthyroid status [110, 111]. TH treatment has also been demonstrated to reverse cognitive deficiencies in rodent models of hypothyroidism [112, 113]. Both mood and cognitive alterations associated with the thyroid status have been attributed to the actions of the THs on some specific structures, particularly the hippocampus. The neuronal population of the hippocampus shows high morphological plasticity that prevails throughout life and is highly sensitive to the stimulation by THs. Animal models of hypo- and hyperthyroidism have shown that thyroid hormones play an essential role in hippocampal neurogenesis [114], synaptogenesis [115, 116], and excitability [117] during adulthood. In previous studies we have observed that adult onset hypothyroidism causes significant changes in the morphology of the CA3 pyramidal cell population, involving neuronal atrophy [118]. Although it has been suggested that these effects of hypothyroidism could be caused by the induction of oxidative stress specifically in the hippocampus [88], the morphological alterations seem to be due primarily to the genomic actions of the THs on the signaling pathways controlling the cell cycle [119]. Finally, most studies in subclinical hypothyroid patients have found no clear detrimental effects attributable to subclinical thyroid disorders on physical, metabolic, and cognitive function in the elder population [120, 121].

Comprehensive studies covering different species have repeatedly found a negative correlation between TH levels and longevity (for review, see [122]). The mechanisms responsible for this age-related decline in serum TSH are still not clearly understood but have been postulated to relate with (1) an apparent resetting of the TH feedback regulation threshold due to an enhanced pituitary conversion of T4 to T3 or increased T4 uptake by the thyrotrophs [123], (2) a primary defect in TH inactivation and disposal that could be related to unchanged serum T4 levels in spite of a reduced tropic drive from pituitary TSH secretion [124, 125], and (3) a progressive decrease in physical activity accompanying senescence or an organic brain disease causing cognitive impairment that could also reduce the TRH secretion from the hypothalamus and other brain areas [126]. In any case, a slightly lower thyroid tone seems to exert some kind of protective context, probably related to reduced oxidative stress [41], that would retard aging and prolong lifespan. Given that THs are necessary for proper cell proliferation, development, and maintenance, their actions on oxidative stress could create a compromised situation in which a tradeoff between proliferation and oxidation is established. The balance between these two aspects would change substantially between the young and the elder because proliferation and growing of the body mass decline and even revert due to biological causes [122]. Therefore, as age progresses the reduction of the metabolic demands along with some drive to reduce catabolism and oxidative stress would push for a reduction in the activity of the thyroid axis. From this perspective the varying degree of activity of the thyroid axis could constitute a potential factor, although probably not a major one, in the development of neurodegenerative diseases and other traits of aging.

## 6. Neurodegenerative Diseases

There are lines of evidence that an alteration on THs levels can modify the progression of a neurodegenerative disease, although there is no evidence of a causal physiopathological link between thyroid status and neurodegenerative diseases. This link does not only comprise THs effects on metabolic activity in general and oxidative stress in particular, but also the widespread mechanisms of action that THs can exert on various cellular pathways.

### 6.1. Alzheimer and THs

Alzheimer's disease (AD) is the most common cause of dementia diagnosed after the age of 60, and it is characterized by neuronal loss as a consequence of neurofibrillary tangles and senile plaques. There are different theories which explain the accumulation of beta amyloid plaques, but abnormal levels of oxidative stress have been reported in both brain and blood stream in AD [127, 128]. An increased oxidatively modified protein and mitochondrial disfunction in AD brain have been reported [129, 130], and it has also been suggested that oxidative stress is a key for the progression of AD [131]. In fact, a wide variety of detectable biomarkers of oxidative stress on biological samples have been proposed to have an opportune diagnosis of AD [132].

There are reports that support that thyroid status is closely related to AD pathogenesis [133, 134]. However, there are data which do not show a clear relation between thyroid status and dementia incidence [135, 136].

Whether a change in TH levels on elderly people is a factor to increase the mobility towards AD in older people is unclear. Numerous works have collected an important number of clinical cases trying to correlate thyroid disorders and dementia. The study by Ceresini and coworkers [137], which analyzed 1171 participants from Italy, showed that thyroid dysfunction tends to be higher in older than in younger persons, with subclinical hyperthyroidism being the most prevalent condition. This study shows an independent association between subclinical hyperthyroidism and cognitive impairment. Its results agree with those obtained by Kalmijn and coworkers [133]. Interestingly, there are studies that demonstrate a very high prevalence of autoimmune thyroid disease in familial Alzheimer's disease [138, 139], while others have reported that the subclinical hypothyroidism state correlates with cognitive impairment in patients aged 65 and over [140]. However, it has also been found that TSH levels are not related to risk of AD, arguing against an important role of thyroid function in the development of AD [141]. As expected in epidemiological studies, there are contradictory results. It is difficult to match the results from different epidemiological studies due to the different criteria of inclusion in each study.

To our knowledge, there is only one study which reports a decrease in mRNA for the thyroid receptor alpha on CA3 and CA1 hippocampal region in Alzheimer brain tissue [142]. A relation between the thyroid hormone receptor alpha gene polymorphisms and AD risk has also been reported [143]. This data is relevant if we consider that triiodothyronine negatively regulates the transcriptional activity of the *β*-amyloid precursor protein (APP) gene in cultures of murine neuroblastoma and rat neurons and in human neuroblastoma [144] with the participation of thyroid hormone receptors [145]. In agreement with these reports, it has been shown that the T4 treatment significantly enhanced the ability in spatial learning and memory task using AD mouse model induced by injection of aggregated beta amyloid into CA1 hippocampal region [146]. These animals showed enhanced cholinergic function and high antioxidant enzymes levels, restored ATP content, and inhibited neuronal apoptosis. The mechanisms of thyroxine treating AD might be associated with regulating the cholinergic function, protecting neurons against the damage from free radicals, and preventing neuronal apoptosis.

An alternative mechanism of TH to improve AD has been described. It proposes that TH could regulate the expression of Seladine-1 (selective AD indicator 1), a gene related to AD [147]. It has been shown that the upregulation of this gene leads to reduction of beta amyloid accumulation. This gene promotes cholesterol synthesis inside the neuron which in turn inhibits colocalization of beta amyloid precursor protein [148]. The increase of the Seladine-1 gene and protein expression in hyperthyroid mice has been proven. Although hypothyroid mice do not show a reduction, they maintain similar levels of the Seladine-1 gene expression to those of euthyroid mice [147].

### 6.2. Multiple Sclerosis and THs

Multiple sclerosis (MS) is a chronic inflammatory disease of the central nervous system characterized mainly as an autoimmune neurodegenerative disorder where phagocytosis and proinflammatory cytokines play a fundamental role. MS is distinguished by the chronic demyelinating of unknown but multiple etiologies. This demyelinating process is accompanied by neuronal and axonal loss; thus, MS is also considered as a neurodegenerative disease. Although it is not totally accepted, studies suggest that oxidative stress may be one of the factors that trigger or exacerbate MS [149, 150]. ROS enhanced migration of monocytes across the blood-brain barrier and oligodendroglial damage have been observed [151, 152]. It has also been observed that oxidative damage in humans is widespread throughout active demyelinating MS lesions, accompanied by an enhanced antioxidant enzyme expression that may be a defense response [153] as well as an increase of oxidative stress in patients with MS [154].

It is widely known that myelin sheaths are produced by oligodendrocytes cells in the central nervous system (CNS). Myelination is a complex process which includes the proliferation and migration of oligodendrocytes, adhesion of oligodendrocytes to the axon, and synthesis of myelin [155]. As a result, there are numerous pathways regulating the myelination process and which are modulated by different signals, as growth factors actually do [156, 157].

The role of THs on myelin formation was documented by Almazan et al. [158], and it has also been proven that the THs therapy could be beneficial for myelination in brains of patients with congenital hypothyroidism [159] or for an experimental model of chronic demyelination [160]. The lack of myelination on neonatal hypothyroidism has been related to the modulation of genes; in fact, a downregulation of proteins related to myelin synthesis persists in adulthood [161, 162]. THs also induce differentiation and maturation of oligodendrocytes *in vitro* [163] and in adult brain [164].

It has been described that autoimmune thyroid disorders are between three and five times more common in MS patients, with woman being with a greater risk of developing them [165, 166]. It is possible that both diseases, hypothyroidism and MS, are a consequence of autoimmune disease. However, due to the importance of THs in myelin formation, the health of the thyroid gland must be taken in to account.

### 6.3. Parkinson's Disease and THs

Parkinson's disease (PD) is a common adult-onset neurodegenerative disorder. It is characterized by the death of dopaminergic neurons of the substantia nigra compacta. This loss of neurons causes shaking, rigidity, slow movement, and damage to the cognitive functions. Its etiology is unknown, although oxidative stress has been linked to both the initiation and the progress of PD [167–169]. An animal model widely used to study PD is performed by i.p. injection of the neurotoxin 1-methyl-4-phenyl-1,2,3,6-tetrahydropyridine (MPTP). Interestingly, it has been observed that both the inflammatory processes and the oxidative stress are related to MPTP-neurodegeneration [170]. The relationship between PD and oxidative stress is not exclusive of animal models; for example, it has been reported that one of the earliest biochemical changes observed in PD patients is the reduction in reduced glutathione [171].

Parkinsonism and thyroid dysfunction have some clinical features. Hypothyroidism can provoke bradykinesia and hypomimia while hyperthyroidism can worsen tremor and dyskinesias [172, 173]. Because of that, the diagnosis of thyroid dysfunction may be difficult in Parkinson's disease patients. However, there is not an apparent pathogenesis relation between thyroid dysfunction and PD. There are few epidemiological studies and they show that there is no evidence of either a high frequency of hypothyroidism [174–176] or thyroid autoimmunity among PD [177].

## 7. Conclusions

Oxidative stress balance is a multifactorial process involving numerous metabolic pathways in the cell. Thyroid hormones play a significant role in ROS production due to their capacity to accelerate the basal metabolism and change respiratory rate in mitochondria. On the other hand, THs also affect the cell antioxidant mechanisms in different ways, thus creating a multivariate situation whose outcome is difficult to predict. The evidence available shows a complex relationship between TH levels and oxidative stress, but the general principle is that elevated TH levels (hyperthyroidism) induce oxidative stress, whereas reduced THs levels (hypothyroidism) result in nondetectable to mild oxidative stress.

The etiology of neurodegenerative diseases is complex, but in all the cases a strong association has been found between aging and oxidative stress. This suggests the participation of THs in the onset and progress of neurodegenerative diseases. It is clear that the thyroid function changes through life span, but the mechanisms and the physiological significance of this modification are not well understood. Moreover, a clear relation between THs and neurodegenerative diseases has not been found. Numerous data in the literature show that changes in TH levels affect the functions of the central nervous system, but the studies reported nowadays indicate that it is difficult to match the onset and progress of neurodegenerative diseases with the thyroid status, probably because of the complex relation between THs and neural performance. The participation of THs on Alzheimer disease is well documented, but the effects of THs are not always explainable as changes in the oxidative stress status.

Finally, it must be considered that some neurodegenerative alterations produce symptoms similar to those of hypothyroid disorders, so that that in some cases underlying thyroid alterations could be masked. It is advisable to check the thyroid status in patients with a neurodegenerative process. The participation of THs in neuronal metabolism is a factor that should not be ruled out when explaining the changes in the elderly brain.

## Figures and Tables

**Table 1:** Effects of hyper- or hypothyroidism on the activity/abundance of antioxidant enzymes and the oxidative status of various tissues.

	Hyperthyroidism								Hypothyroidism							
Tissue	SOD	GPx	CAT	GSH	Lpx	Chl	Crb	Species	SOD	GPx	CAT	GSH	Lpx	Chl	Crb	Species
Heart	↑	↓	↓		↑			Rat [18]	↓				—			Rat [18]
	↑	↑	—	↓	↑	↑	↑	Rat [48]	↓	↓	↓		↓		↓	Rat [41]
		—			↑			Rat [39]		↑			—			Rat [39]
				↑	↑			Rat [49]				↓	↓			Rat [49]
						↑		Rat [50]	—	—	—		—			Rat young [42]
	↑	—	—		—			Rat young [42]	—	—	—		—			Rat old [42]
	—	↓	—		↑			Rat old [42]								

Liver					↓			Mouse [51]					—			Mouse [51]
	—	↓	↓		—			Rat [18]	—				—			Rat [18]
	↓		↓			↑		Rat [40]	—	↑	—		—			Rat [52]
	↑	↑	↑		↑↑			Rat [52]					↓		↓↓	Rat [53]
					↓		↓↓	Rat [53]		—			—			Rat [39]
		↑					↑	Rat [54]				↓	↓			Rat [49]
		—			↑			Rat [39]								
				↑	↑			Rat [49]								
	↓	↓		↓	↑		↑	Rat [55]								
						↑		Rat [50]								
	↓	↓	↓	↓				Rat [56]								
					↑		↑	Rat [57]								
		↑		↓	↑			Rat [58]								
*Microsomes *					↑			Rat [32]								
*Mitochondria *							↑	Rat [54]								

Skeletal muscle					↑			Mouse [35]	↓	—			↑			Duck [59]
		↑			↑			Rat [39]					—			Mouse [35]
										↑			—			Rat [39]
*Oxidative *	↑	↓	↓		↑			Rat [18]	↓	—	—		—			Rat [18]
*Glycolytic *									↓	—	—		—			Rat [18]

Blood																
*Plasma *					↑			Rat [60]	↓				↑			Rat [61]
						↑		Rat [50]	↓	↑	—	↓	↑			Human [62]
		—		↑	↑			Rat [63]	—		↑		↑			Human [64]
					↑			Human [65]					↑			Human [66]
					↑			Human [67]	↑		↑		↑			Human [68]
					↑			Human [69]					—			Human [70]
					↑			Human [71]		↑	—	↓	↑↑		↑	Human [72]
	↓				↑			Human [73]					↑		↑	Human [74]
	↑	↓	↑		↑			Human [75]					↑		↑	Human [76]
					↑			Human [66]	↓	↓	↓					Human [77]
					↑			Human [78]					↑			Human [78]
*Erythrocytes *	—		—		↑		↑	Rat [79]	—		—		↓		↓	Rat [79]
	↑			↓	↑			Rat [80]								
	↑	↓	—		—			Rat [18]								
				—				Cat [81]								
	↑	↑			—			Human [73]								

Brain																
*Homogenate *				↑	↑			Rat [49]				↓	↓			Rat [49]
	↑	↑	↑			↑↑		Rat juvenile [27]	↑		↑	↓	↑		↑	Rat neonate [82]
	↓	↓	↑		↑			Rat neonate [83]	↓↓	↓	↓		↑			Rat neonate [83]
*Mitochondria *	—	↓			↑		↑↑	Rat [19]	↑	↑			↑		↑	Rat [19]
*Cortex *					↑↑			Rat [19]					—			Rat [19]
	↑				↑			Rat [84]	—				—			Rat [85]
	—	↑	↑		—			Rat old [86]	↓	↑	↓		↓			Rat neonate [87]
									↑	↑	—		↑			Rat old [86]
*Hippocampus *									↓				↑			Rat [61]
													↑			Rat [88]
												↓	↑			Rat neonate [89]
*Cerebellum *	↓				—			Rat [84]	↓				↑			Rat [85]
													—			Rat [88]
	↓↓	↓	↓		↑			Rat neonate [83]	↓	—	↑		↓			Rat juvenile [90]
									↑	—	—		↑			Rat neonate [90]
									↓↓	↓	↓		↑			Rat neonate [83]
*Medulla *	↓↓	↓	↓		↑			Rat neonate [83]	↓↓	↓	↓		↑			Rat neonate [83]

Antioxidant enzymes and substrates: SOD: superoxide dismutase (no distinction is made between Cu/Zn-SOD and Mn-SOD); GPx: glutathione peroxidase; CAT: catalase; GSH: reduced glutathione.

Oxidative status: Lpx: lipid peroxidation (measured as thiobarbituric acid-reactive substances: TBARS or malondialdehyde production); Chl: chemiluminescence; Crb: carbonylated proteins.

↓: decrease; ↑: increase; —: no change. Double arrows represent a highly significant effect (*P* < 0.001).
